# Optimal density-functional theory method for zinc–amino acid complexes determined by analyzing structural and Fourier-transform infrared spectroscopy data†

**DOI:** 10.1039/d3ra07172c

**Published:** 2024-01-02

**Authors:** Unghwi Yoon, Jongsik Kim, Sang Hoon Kim, Keunhong Jeong

**Affiliations:** a Extreme Materials Research Center, Korea Institute of Science and Technology (KIST) Seoul 02792 South Korea kim_sh@kist.re.kr; b Division of Nano & Information Technology at KIST School, University of Science and Technology (UST) Daejeon 34113 South Korea; c Department of Chemical Engineering, Kyung Hee University Yongin 17104 South Korea; d Department of Physics & Chemistry, Korea Military Academy (KMA) Seoul 01805 South Korea doas1mind@kma.ac.kr

## Abstract

Metal–amino acid complexes are important compounds for the human body. Their nutritional value and anticancer, antibacterial, and catalytic properties are the focus of several studies. Density functional theory (DFT) can be used to predict their properties by optimizing their structures and performing electron population analyses. However, conventional computational methods cannot adequately determine the parameters of polymeric metal–amino acid complexes. Therefore, intermolecular interactions of polymers must be considered to correctly predict the properties of metal–amino acid and related metal complexes. In this study, different DFT protocols were used to acquire the infrared spectra and determine interatomic distances of two zinc–amino acid complexes, Zn(Gly)_2_ and Zn(Met)_2_. The results were compared to spectroscopic and X-ray crystallographic data, revealing that the M06 and M06-L functionals and the 6-311++G(d,p) basis set produced the smallest computational errors. Our results provide a foundation for future theoretical studies on other metal–amino acid and metal–organic complexes.

## Abstract 2

Metal–amino acid complexes, vital for health, pose computational analysis challenges. This study employs DFT to accurately analyze zinc–amino acid complexes, guiding future metal–organic complex research.

## Graphical abstract

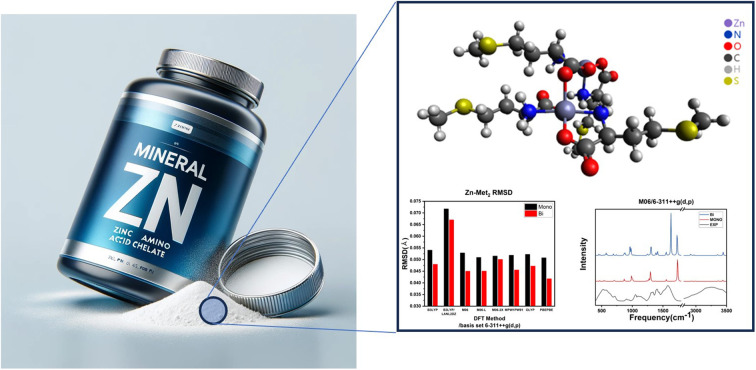

## 1. Introduction

Zinc is an essential trace element in animals and is present in many metabolic and signaling pathways within the human body.^1^ Zinc deficiency often causes health problems such as malabsorption of nutrients, growth retardation, and a compromised immune system.^2,3^ In 2017, one billion people worldwide were reported to suffer from chronic zinc deficiency.^4^ In nature, zinc exists in mineral salts, metal chelates, and amino acid chelates.^5^ Amino acid chelates are soluble low-molecular-weight compounds that can significantly improve the bioavailability of zinc.^6^ In zinc–amino acid coordination compounds, the oxygen atom of the carboxylate group and the unshared electron pair of the nitrogen atom bind to the metal to form a chelate. Coordination complexes of amino acids and metals have been actively studied because of their involvement in nutrition, anticancer and antibacterial processes, and catalytic activities in the human body. Investigation of the structures of Zn(ii)–amino acid complexes can promote the development of new nutritional compounds.^7–11^ Quantum chemical calculations also provide important information regarding the formation and bonding of Zn(ii)–amino acid complexes through *in silico* study of their properties.

**Fig. 1:**
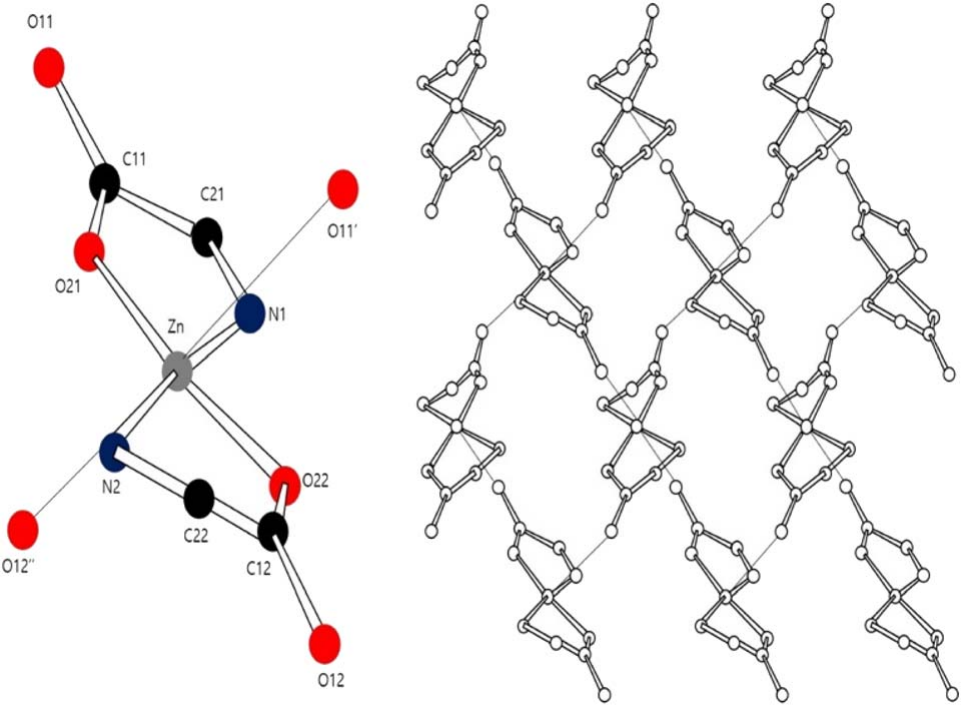
Coordination environment of the Zn center in a Zn–Met_2_ crystal.

Density functional theory (DFT) has been used to investigate the application of metal–amino acid complexes in diverse fields. Rulisek and Havlas^12^ studied the ion selectivity of transition metals and amino acids by comparing their interaction energies. Dudev and Lim^13^ examined the affinity and selectivity of metal cofactors in proteins by comparing the binding free energies of amino acid residues and metals *via* DFT calculations. Berestova *et al.*^14^ studied the *cis* and *trans* isomers of homo- and hetero-amino acid complexes of copper using infrared (IR) spectroscopy. The accuracy of DFT calculations is typically assessed by comparing the computed bond lengths and angles with experimental values. However, metal–amino acid complexes in the solid state often exist in the form of polymer crystals comprising several molecules whose interactions cannot be ignored (Fig. 1 (ref. 15 and 16)).

**Fig. 2:**
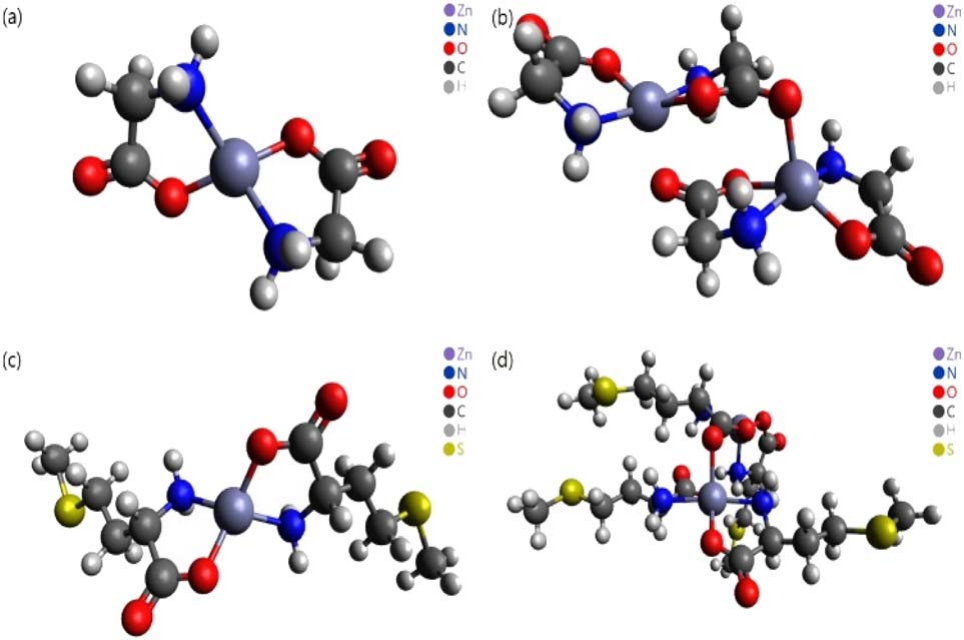
Molecular structures optimized using the M06 functional. (a) Zn–Gly_2_ single molecule, (b) Zn–Gly_2_ double molecule, (c) Zn–Met_2_ single molecule, and (d) Zn–Met_2_ double molecule.

In this study, different DFT protocols were evaluated by comparing the calculated bond lengths and IR spectra of zinc–glycine and zinc–methionine complexes with experimental values. To illustrate the diverse coordination environments of zinc, we present both the monomeric (Fig. 2a and c) and dimeric (Fig. 2b and d) forms of these complexes, while ESI Fig. S1† shows the generalized repeating unit structures. This distinction highlights the adaptability of zinc in different molecular contexts—from the typical four-ligand coordination in monomers to the more complex five-ligand coordination in dimers—which is essential for interpreting our computational and experimental findings. Double and single molecules were simulated to account for the polymerization of the complexes. Six different DFT methods were employed using the 6-311++G(d,p) basis set. A basis set based on the triple zeta quality effective core potential (ECP) such as LANL2TZ+ is typically recommended for DFT calculations of transition metals^17^ However, previous studies^18–21^ showed that calculations of dual zeta cores such as 6-311++G(d,p) are suitable for transition metal ligands. The accuracy of the 6-311++G(d,p) basis set was verified by comparing the results of B3LYP/6-311++G(d,p) and B3LYP/LanL2DZ calculation.^14^ As a result, the 6-311++G(d,p) basis set was proven more suitable that the LanL2DZ basis set for calculating the interatomic length of Zn-Amino acid molecule and IR spectrum. Calculations employing the M06 and M06-L DFT methods with the 6-311++G(d,p) basis set produced the smallest errors relative to the experimental values. Both single (monomer) and double molecular (dimer) models were used to identify the most accurate representation of the polymeric zinc–amino acid complexes. Herein, we describe the optimum DFT method to simulate zinc–amino acid complexes and report that their optimized geometries are in close agreement with experimental parameters.

## 2. Experimental

### 2.1 Synthesis of zinc–glycine and zinc–methionine chelates and measurement of their vibrational spectra

The experimental setup comprised a 2-L jacketed crystallizer, an electric stirrer, and a thermostat (Polyscience, AP07R-20). Methionine or glycine (0.4 mol) and sodium hydroxide (0.4 mol) were dissolved in distilled water (1000 mL), injected into the crystallizer, and stirred at a speed of 400 rpm and a constant temperature (30 °C) controlled using the thermostat. Zn–amino acid nucleation was induced by injecting an aqueous ZnCl_2_ solution (30 °C, 2 M, 100 mL) into the aqueous amino acid/hydroxide solution using a peristaltic pump (Ismatec, Reglo, ICC pump, Wertheim). The Zn–amino acid crystals were recovered through vacuum filtration and dried in a vacuum oven at 40 °C for 24 h. Vibrational spectra were recorded using attenuated total reflectance (ATR)–Fourier-transform infrared (FTIR) spectroscopy (React-IR 15, Mettler Toledo) over a frequency region of 3500–400 cm^−1^ at a 4 cm^−1^ resolution.

### 2.2 DFT calculation

The Zn–amino acid complexes were studied employing the B3LYP,^22,23^M06,^24^ M06-L,^25,26^ M06-2X,^24^ MPW1PW91,^27^ OLYP,^28^ and PBEPBE^29^ DFT functionals with the Gaussian 16 software and GaussianView6 (Fig. 2).^30,31^ The molecular structure was visualized using the Avogadro software.^32^ The B3LYP functional along with the 6-311++G(d,p)^33^ and LanL2DZ^34^ basis sets were used to calculate the minimum energies and bond lengths. The other functionals were used only with the 6-311++G(d,p) basis set. Experimental and simulated FTIR spectra were employed to identify the stretching and bending vibrations of the complexes.

## 3. Results & discussion

### 3.1 Bond length deviations obtained using various calculation DFT methods

**Fig. 3:**
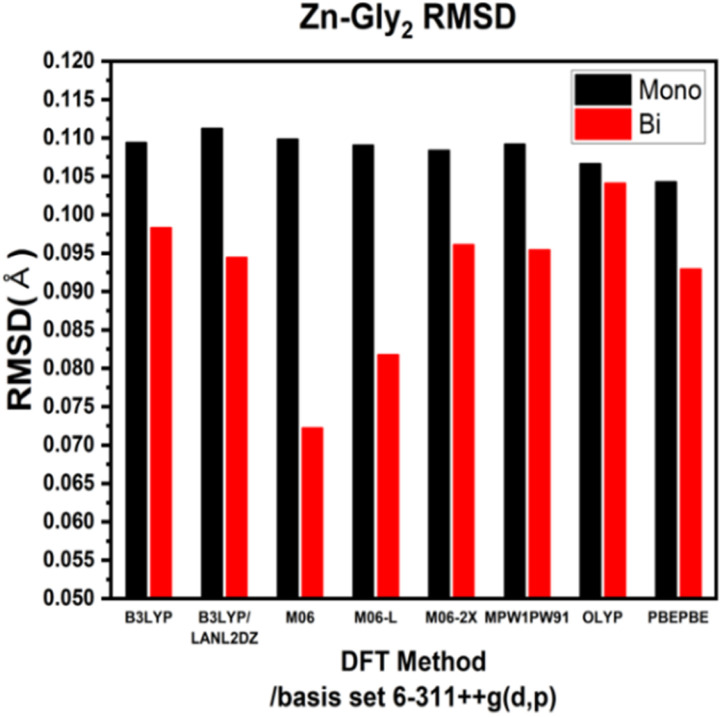
RMSDs of the theoretical *versus* the experimental bond lengths of single and double Zn–Gly_2_.

**Fig. 4:**
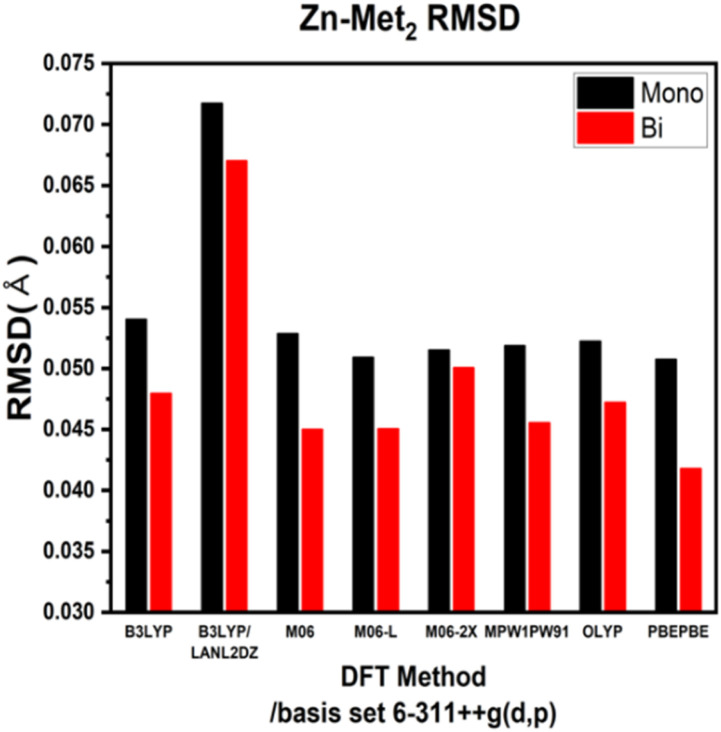
RMSDs of the theoretical *versus* the experimental bond lengths of single and double Zn–Met_2_.

ESI Tables S1–S4† show the comparison of the interatomic bond lengths of the zinc–glycine and zinc–methionine complexes obtained using X-ray crystallography^13,14^ and those obtained using DFT calculations. Fig. 3 and 4 and ESI Tables S5 and S6† list the root-mean-square deviations (RMSDs) of the experimental and calculated parameters. The smallest RMSDs are obtained using the 6-311++G(d,p) basis set, whereas the results obtained for double Zn–Gly_2_ (Fig. 4) are an outlier. Notably, the RMSDs of double Zn–Gly_2_ and Zn–Met_2_ are smaller than those of the corresponding single molecules in all calculations (red bars in Fig. 3 and 4). For single Zn–Gly_2_, the bond lengths computed using PBEPBE are the closest to the experiment values; however, the M06 functional provided the most accurate results for double Zn–Gly_2_ (Fig. 3). For Zn–Gly_2_, the largest difference between the single and double molecules (0.0377 Å) is observed using M06, whereas the smallest difference is obtained using OLYP (0.0026 Å). The bond lengths computed using the PBEPBE functional are closest to the experiment values for the single and double Zn–Met_2_ molecules (Fig. 4). For Zn–Met_2_, the largest difference between the single and double molecules (0.0090 Å) is obtained using PBEPBE, whereas the smallest difference (0.0015 Å) is obtained using M06-2X. Overall, the differences between the single and double molecules of zinc–methionine are smaller than those of zinc–glycine.

### 3.2 Calculated vibrational spectra and asymmetric –COO stretching bands

**Fig. 5:**
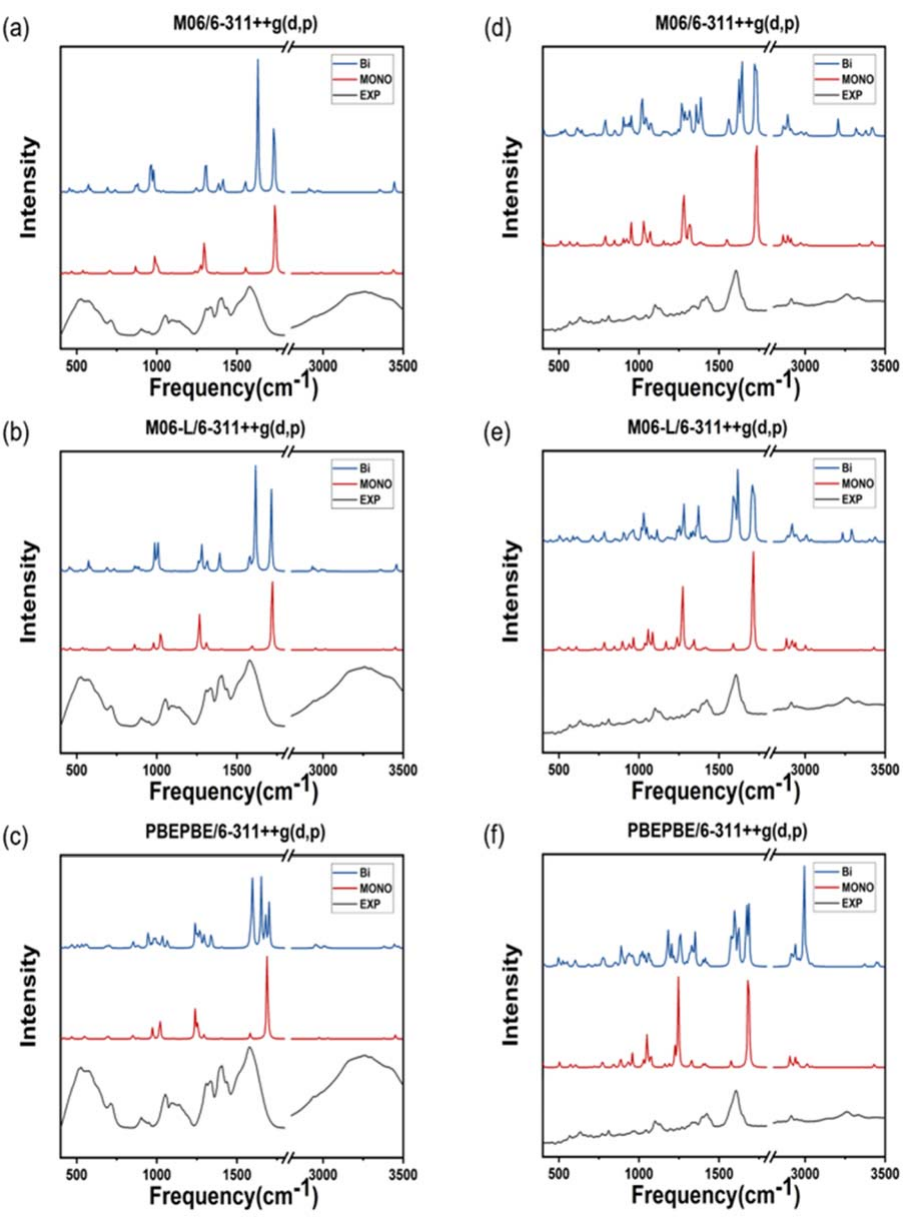
IR spectra of Zn–Gly_2_ calculated using the (a) M06, (b) M06-L, and (c) PBEPBE functionals. IR spectra of Zn–Met_2_ calculated using the (d) M06, (e) M06-L, and (f) PBEPBE functionals. The experimental spectrum obtained using ATR-FTIR spectroscopy is included for reference.

The theoretical IR spectra obtained *via* quantum calculations were compared to the experimental spectra after multiplication by a scaling factor (Tables 1 and 2). ATR-FTIR spectroscopy is typically performed to analyze a solvent in a solution; however, in the case of metal–amino acid chelates, the insoluble suspended solids are predominantly analyzied.^35^ Consequently, the peak vibrational frequencies calculated for dimeric molecules reflect the experimental data more accurately compared with those computed for monomeric molecules. The purpose of this study was to identify the most accurate protocol for computing the properties of two different metal complexes as polymer crystals (Fig. 5). Most peaks calculated in the IR spectra of double Zn(Gly)_2_ are identical to those of single Zn(Gly)_2_. However, regions of the double molecule, which are in close proximity to its partner, yield peaks at different energies than those of the single molecule. This finding is attributed to the polarization of the two units in the molecular pair. The vibrational frequencies arising from regions remote from the molecular partner are similar to those of the single molecule. Thus, the IR spectra of the double molecules include characteristic peaks from both the single molecule and the polymerized one. The IR spectrum of a solid can be therefore simulated by taking these interactions into account within an appropriate structural model. Tables 1 and 2 list the vibrational frequencies of the asymmetric –COO stretching band, corresponding to the most intense peaks of the single and double models. The B3LYP/LanL2DZ combination produces the smallest difference between the calculated and experimental vibrational frequencies of both the Zn–Gly_2_ and Zn–Met_2_ single molecules. However, the smallest differences between the calculated and experimental frequencies for double Zn–Gly_2_ and Zn–Met_2_ occur when using the M06-L DFT method.

**Table 1:** Vibrational frequencies of the asymmetric –COO stretching band of Zn–Gly_2_ calculated for single and double molecules

DFT method	Zn–(Gly)_2_*ν*(C=O) characteristic IR peaks (cm^−1^)/6-311++G(d,p)		
	Scale factor	Mono	Bi
B3LYP	0.964	1693	1655
B3LYP/LanL2DZ	0.961	1610	1557
M06	0.96	1739	1630
M06-L	0.952	1719	1615
M06-2X	0.96	1745	1696
MPW1PW91	0.957	1723	1684
OLYP	0.9839	1694	1665
PBEPBE	0.99	1688	1651
Experimental		1593	

**Table 2:** Vibrational frequencies of the asymmetric –COO stretching band of Zn–Met_2_ calculated for single and double molecules

DFT method	Zn–(Met)_2_*ν*(C=O) characteristic IR peaks (cm^−1^)/(6-311++G(d,p))		
	Scale factor	Mono	Bi
B3LYP	0.964	1687	1630
B3LYP/LanL2DZ	0.961	1600	1518
M06	0.96	1733	1622
M06-L	0.952	1712	1618
M06-2X	0.96	1737	1627
MPW1PW91	0.957	1715	1646
OLYP	0.9839	1690	1635
PBEPBE	0.99	1683	1621
Experimental		1606	

## 4. Conclusions

The bond lengths of Zn–Gly_2_ and Zn–Met_2_ calculated using different DFT protocols were compared to experimental values determined using X-ray crystallography. The RSMD differences between the single and double molecules of both compounds were in the range of 0.03–0.005 Å. Compared to single molecules, the calculated bond lengths of double molecules were more accurate because the double molecules resemble the polymerized forms of zinc–amino acid complexes more closely. Based on the RMSDs of the double molecule models, the bond lengths closest to the experimental values were obtained using the M06, M06-L, and PBEPBE functionals for Zn–Gly_2_ (RMSDs: 0.0722, 0.0818, and 0.0930 Å, respectively) and the PBEPBE, M06, and M06-L functionals for Zn–Met_2_ (RMSDs: 0.0418, 0.045, and 0.0451 Å, respectively). The calculated frequencies of the most intense asymmetric –COO stretching bands revealed that the energies determined using M06-L (1618 cm^−1^) exhibited the smallest deviations from the experimental values (1606 cm^−1^). These results indicate that an optimal computational protocol can be determined based on IR vibrational frequencies, bond lengths, and bond angles. Such methodology can be applied to metal–amino acid complexes other than Zn–Gly_2_ and Zn–Met_2_ because metal chelates generally possess comparable properties. The results of this study may be used to elucidate the physiological absorption mechanisms of metal–amino acid complexes *via* theoretical calculations to investigate their anticancer and antibacterial properties.

## Author contributions

We Unghwi Yoon: writing – original draft preparation, visualization, investigation; Jonsik Kim: reviewing; Sanghoon Kim: reviewing, supervision; Keunhong Jeong: supervision, date curation, writing, and editing.

## Conflicts of interest

There are no conflicts to declare.

## Supplementary Material

RA-014-D3RA07172C-s001
