# Supplementary material for: Optimal density-functional theory method for zinc–amino acid complexes determined by analyzing structural and Fourier-transform infrared spectroscopy data
Source: RSC Adv. 2024 Jan 2;14(2):1051–5. doi: 10.1039/d3ra07172c (PMC10759963; doi:10.1039/d3ra07172c)
Supplement: RA-014-D3RA07172C-s001 [file RA-014-D3RA07172C-s001.pdf]

Electronic Supplementary Material (ESI) for RSC Advances.
This journal is © The Royal Society of Chemistry 2023

Supporting Information

Optimal density-functional theory method for zinc–amino acid complexes determined by analyzing structural and Fourier-transform infrared spectroscopy data

Unghwi Yoon[a,b], Jongsik Kim[c], Sang Hoon Kim[a,b]*, Keunhong Jeong[d]*

[a]Extreme Materials Research Center, Korea Institute of Science and Technology (KIST), Seoul 02792, South Korea.

[b]Division of Nano & Information Technology at KIST School, University of Science and Technology (UST), Daejeon 34113, South Korea.

[c]Department of Chemical Engineering, Kyung Hee University, Yongin 17104, South Korea

[d]Department of Physics & Chemistry, Korea Military Academy (KMA), Seoul 01805, South Korea.

Corresponding Author : S.H. Kim : kim_sh@kist.re.kr
K. Jeong : doas1mind@kma.ac.kr

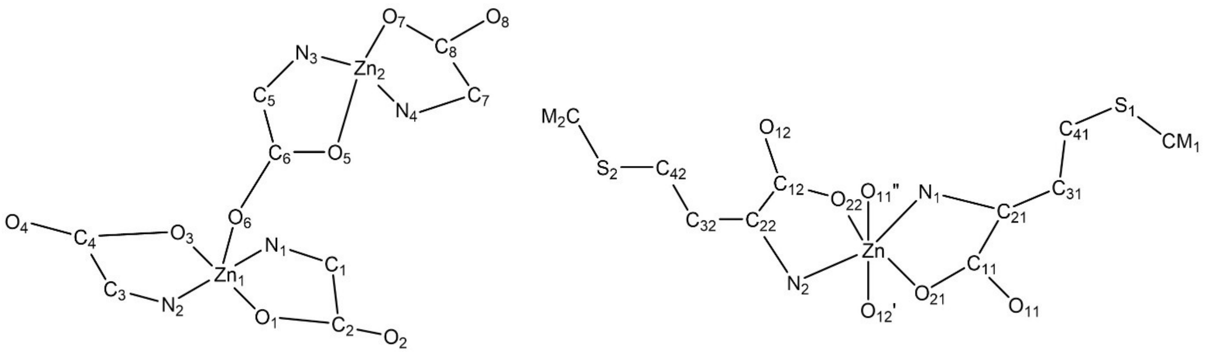

SI Fig. 1. Basic structure of Zn-Gly_2 and Zn-Met_2

SI Table 1. Calculated bond lengths of single Zn-Gly_2.

	**Bond Length(Å) /6-311++G(d,p)**				
**Bond**	**Experiment**	**B3LYP/ LANL2DZ**	**B3LYP**	**M06**	**M06-L**
Zn1–N1	2.023	2.159	2.134	2.104	2.131
Zn1–N2	2.035	2.159	2.135	2.103	2.131
Zn1–O1	2.114	1.938	1.922	1.907	1.920
Zn1–O3	2.174	1.938	1.922	1.907	1.920
C2–O1	1.271	1.333	1.302	1.293	1.300
C2–O2	1.242	1.248	1.214	1.208	1.214
C1–N1	1.476	1.513	1.495	1.484	1.488
C4–O3	1.252	1.333	1.302	1.293	1.300

C4–O4	1.265	1.248	1.214	1.208	1.214
C3–C4	1.516	1.562	1.553	1.542	1.542
C3–N2	1.475	1.513	1.495	1.484	1.488
**Bond**	**M06-2X**	**MPW1PW9**	**OLYP**	**PBEPBE**	
Zn1–N1	2.133	2.111	2.157	2.126	
Zn1–N2	2.134	2.111	2.157	2.126	
Zn1–O1	1.924	1.911	1.942	1.933	
Zn1–O3	1.923	1.911	1.943	1.933	
C2–O1	1.295	1.295	1.308	1.312	
C2–O2	1.208	1.210	1.222	1.224	
C1–N1	1.488	1.483	1.495	1.497	
C4–O3	1.295	1.295	1.308	1.312	
C4–O4	1.208	1.210	1.222	1.224	
C3–C4	1.550	1.544	1.558	1.555	
C3–N2	1.488	1.483	1.495	1.497	

SI Table 2. Calculated bond lengths of single Zn-Met_2

	**Bond Length(Å) /6-311++G(d,p)**				
**Bond**	**Experiment**	**B3LYP/ LANL2DZ**	**B3LYP**	**M06**	**M06-L**
Zn-O21	2.059	1.939	1.922	1.908	1.921
Zn-O22	2.049	1.939	1.922	1.908	1.921
Zn-N1	2.052	2.145	2.118	2.089	2.114

Zn-N2	2.064	2.145	2.119	2.089	2.114
C11-O11	1.25	1.25	1.21	1.21	1.21
C11-O21	1.25	1.33	1.30	1.29	1.30
C11-C21	1.57	1.58	1.57	1.56	1.56
C21-N1	1.48	1.52	1.50	1.49	1.50
C21-C31	1.53	1.54	1.53	1.52	1.52
C31-C41	1.53	1.54	1.53	1.52	1.52
C41-S1	1.79	1.90	1.84	1.82	1.82
S1-C(M1)	1.76	1.89	1.82	1.81	1.80
C12-O12	1.24	1.25	1.21	1.21	1.21
C12-O22	1.28	1.33	1.30	1.29	1.30
C12-C22	1.53	1.58	1.57	1.56	1.56
C22-N2	1.48	1.52	1.50	1.49	1.50
C22-C32	1.55	1.54	1.53	1.52	1.52
C32-C42	1.51	1.54	1.53	1.52	1.52
C42-S2	1.81	1.90	1.84	1.82	1.82
S2-C(M2)	1.79	1.89	1.82	1.81	1.80
**Bond**	**M06-2X**	**MPW1PW9**	**OLYP**	**PBEPBE**	
Zn-O21	1.924	1.911	1.941	1.933	
Zn-O22	1.924	1.911	1.941	1.933	
Zn-N1	2.121	2.096	2.137	2.109	
Zn-N2	2.121	2.096	2.137	2.109	
C11-O11	1.21	1.21	1.22	1.22	
C11-O21	1.29	1.29	1.31	1.31	

C11-C21	1.56	1.56	1.58	1.57	
C21-N1	1.49	1.49	1.50	1.50	
C21-C31	1.52	1.52	1.54	1.53	
C31-C41	1.52	1.52	1.53	1.53	
C41-S1	1.82	1.82	1.84	1.83	
S1-C(M1)	1.81	1.81	1.82	1.82	
C12-O12	1.21	1.21	1.22	1.22	
C12-O22	1.29	1.29	1.31	1.31	
C12-C22	1.56	1.56	1.58	1.57	
C22-N2	1.49	1.49	1.50	1.50	
C22-C32	1.52	1.52	1.54	1.53	
C32-C42	1.52	1.52	1.53	1.53	
C42-S2	1.82	1.82	1.84	1.83	
S2-C(M2)	1.81	1.81	1.82	1.82	

SI Table 3. Calculated bond lengths of double Zn-Gly_2

**Bond Length(Å) /6-311++G(d,p)**					
**Bond**	**Experiment**	**B3LYP/ LANL2DZ**	**B3LYP**	**M06**	**M06-L**
Zn1–O6	2.011	2.107	2.111	2.081	2.101
Zn1–N1	2.023	2.178	2.186	2.123	2.145
Zn1–N2	2.035	2.187	2.196	2.127	2.148
Zn1–O1	2.114	2.053	1.979	1.947	2.125

Zn1–O3	2.174	1.986	2.009	2.118	1.957
Zn2–N4	1.973	2.093	2.119	2.122	2.147
Zn2–N3	2.070	2.18	2.106	2.126	2.142
Zn2–O7	2.130	1.929	1.913	1.946	1.954
Zn2–O5	2.160	2.003	1.959	2.111	2.116
C7–N4	1.474	1.511	1.5	1.477	1.482
C2–O1	1.271	1.308	1.295	1.288	1.265
C2–O2	1.242	1.273	1.219	1.211	1.256
C1–N1	1.476	1.493	1.486	1.478	1.468
C4–O3	1.252	1.326	1.294	1.288	1.295
C4–O4	1.265	1.253	1.225	1.211	1.217
C3–C4	1.516	1.561	1.549	1.541	1.542
C3–N2	1.475	1.502	1.482	1.477	1.482
C6–O5	1.238	1.302	1.276	1.257	1.265
C6–O6	1.271	1.276	1.243	1.252	1.256
C5–C6	1.512	1.543	1.539	1.528	1.528
C5–N3	1.463	1.502	1.492	1.464	1.468
C8–O7	1.264	1.342	1.304	1.288	1.295
C8–O8	1.243	1.247	1.212	1.211	1.217
C7–C8	1.515	1.561	1.553	1.541	1.542
**Bond**	**M06-2X**	**MPW1PW9**	**OLYP**	**PBEPBE**	
Zn1–O6	2.075	2.088	2.208	2.128	
Zn1–N1	2.176	2.160	2.207	2.167	
Zn1–N2	2.198	2.170	2.209	2.177	

Zn1–O1	1.977	1.969	1.998	1.994	
Zn1–O3	2.009	1.998	2.023	2.021	
Zn2–N4	2.121	2.097	2.142	2.111	
Zn2–N3	2.107	2.084	2.132	2.097	
Zn2–O7	1.915	1.902	1.933	1.924	
Zn2–O5	1.959	1.947	1.977	1.970	
C7–N4	1.491	1.487	1.499	1.501	
C2–O1	1.289	1.288	1.301	1.305	
C2–O2	1.213	1.215	1.227	1.229	
C1–N1	1.480	1.475	1.487	1.489	
C4–O3	1.287	1.288	1.301	1.305	
C4–O4	1.221	1.221	1.231	1.235	
C3–C4	1.544	1.540	1.557	1.553	
C3–N2	1.477	1.471	1.483	1.484	
C6–O5	1.268	1.270	1.286	1.287	
C6–O6	1.239	1.238	1.246	1.253	
C5–C6	1.534	1.529	1.546	1.539	
C5–N3	1.485	1.481	1.492	1.494	
C8–O7	1.297	1.297	1.311	1.314	
C8–O8	1.206	1.208	1.220	1.222	
C7–C8	1.550	1.544	1.558	1.555	

SI Table 4. Calculated bond lengths of double Zn-Met_2

Bond Length(Å)

/6-311++G(d,p)					
**Bond**	**Experiment**	**B3LYP/ LANL2DZ**	**B3LYP**	**M06**	**M06-L**
Zn-O21	2.059	2.075	2.033	2.046	2.062
Zn-O22	2.049	1.982	1.984	1.950	1.964
Zn-O11"	2.240	2.150	2.145	2.130	2.141
Zn-N1	2.052	2.183	2.180	2.154	2.176
Zn-N2	2.064	2.147	2.143	2.104	2.120
C11-O11	1.25	1.27	1.24	1.23	1.24
C11-O21	1.25	1.32	1.28	1.28	1.28
C11-C21	1.57	1.56	1.56	1.54	1.54
C21-N1	1.48	1.51	1.49	1.48	1.48
C21-C31	1.53	1.54	1.53	1.52	1.52
C31-C41	1.53	1.54	1.53	1.52	1.52
C41-S1	1.79	1.90	1.84	1.82	1.82
S1-C(M1)	1.76	1.89	1.83	1.81	1.81
C12-O12	1.24	1.25	1.22	1.21	1.22
C12-O22	1.28	1.33	1.30	1.29	1.30
C12-C22	1.53	1.57	1.56	1.55	1.56
C22-N2	1.48	1.51	1.49	1.48	1.49
C22-C32	1.55	1.54	1.54	1.52	1.52
C32-C42	1.51	1.54	1.53	1.52	1.52
C42-S2	1.81	1.90	1.84	1.82	1.82
S2-C(M2)	1.79	1.89	1.83	1.81	1.80

**Bond**	**M06-2X**	**MPW1PW9**	**OLYP**	**PBEPBE**	
Zn-O21	2.056	2.022	2.044	2.045	
Zn-O22	1.967	1.967	1.996	1.989	
Zn-O11"	2.118	2.124	2.242	2.174	
Zn-N1	2.187	2.162	2.201	2.164	
Zn-N2	2.133	2.118	2.159	2.122	
C11-O11	1.24	1.24	1.24	1.25	
C11-O21	1.27	1.27	1.28	1.29	
C11-C21	1.55	1.55	1.57	1.56	
C21-N1	1.48	1.47	1.49	1.49	
C21-C31	1.53	1.52	1.54	1.53	
C31-C41	1.52	1.52	1.53	1.53	
C41-S1	1.82	1.82	1.84	1.84	
S1-C(M1)	1.81	1.81	1.82	1.82	
C12-O12	1.21	1.22	1.23	1.23	
C12-O22	1.29	1.29	1.30	1.30	
C12-C22	1.56	1.56	1.58	1.57	
C22-N2	1.48	1.48	1.49	1.49	
C22-C32	1.53	1.52	1.54	1.53	
C32-C42	1.52	1.52	1.53	1.53	
C42-S2	1.82	1.82	1.84	1.84	
S2-C(M2)	1.81	1.81	1.82	1.82	

SI Table 5. RMSD between literature values and values calculated by DFT for Zn-Gly_2. Rank

determined in ascending order of RMSD.

Zn-Gly_2 RMSD / basis set 6-311++g(d,p)					
**Method**		**B3LYP**	**B3LYP/Lanl2dz**	**M06**	**M06-L**
Mono	RMSD	0.109435	0.111272	0.109905	0.109093
	Rank	6	8	7	4
**Method**		**M06-2X**	**MPW1PW91**	**OLYP**	**PBEPBE**
Mono	RMSD	0.108418	0.109257	0.106706	0.104346
	Rank	3	5	2	1
**Method**		**B3LYP**	**B3LYP/Lanl2dz**	**M06**	**M06-L**
Bi	RMSD	0.098368	0.094459	0.072247	0.081803
	Rank	7	4	1	2
**Method**		**M06-2X**	**MPW1PW91**	**OLYP**	**PBEPBE**
Bi	RMSD	0.096145	0.095448	0.104142	0.09297
	Rank	6	5	8	3

SI Table 6. RMSD between literature values and values calculated by DFT for Zn-Met_2. Rank determined in ascending order of RMSD.

Zn-Met_2 RMSD / basis set 6-311++g(d,p)				
**Method**	**B3LYP**	**B3LYP/Lanl2dz**	**M06**	**M06-L**

Mono	RMSD	0.054059	0.071756	0.05288	0.050952
	Rank	7	8	6	2
**Method**		**M06-2X**	**MPW1PW91**	**OLYP**	**PBEPBE**
Mono	RMSD	0.051541	0.051888	0.052277	0.050784
	Rank	3	4	5	1
**Method**		**B3LYP**	**B3LYP/Lanl2dz**	**M06**	**M06-L**
Bi	RMSD	0.047993	0.067048	0.045015	0.045081
	Rank	6	8	2	3
**Method**		**M06-2X**	**MPW1PW91**	**OLYP**	**PBEPBE**
Bi	RMSD	0.050089	0.045587	0.047251	0.041804
	Rank	7	4	5	1

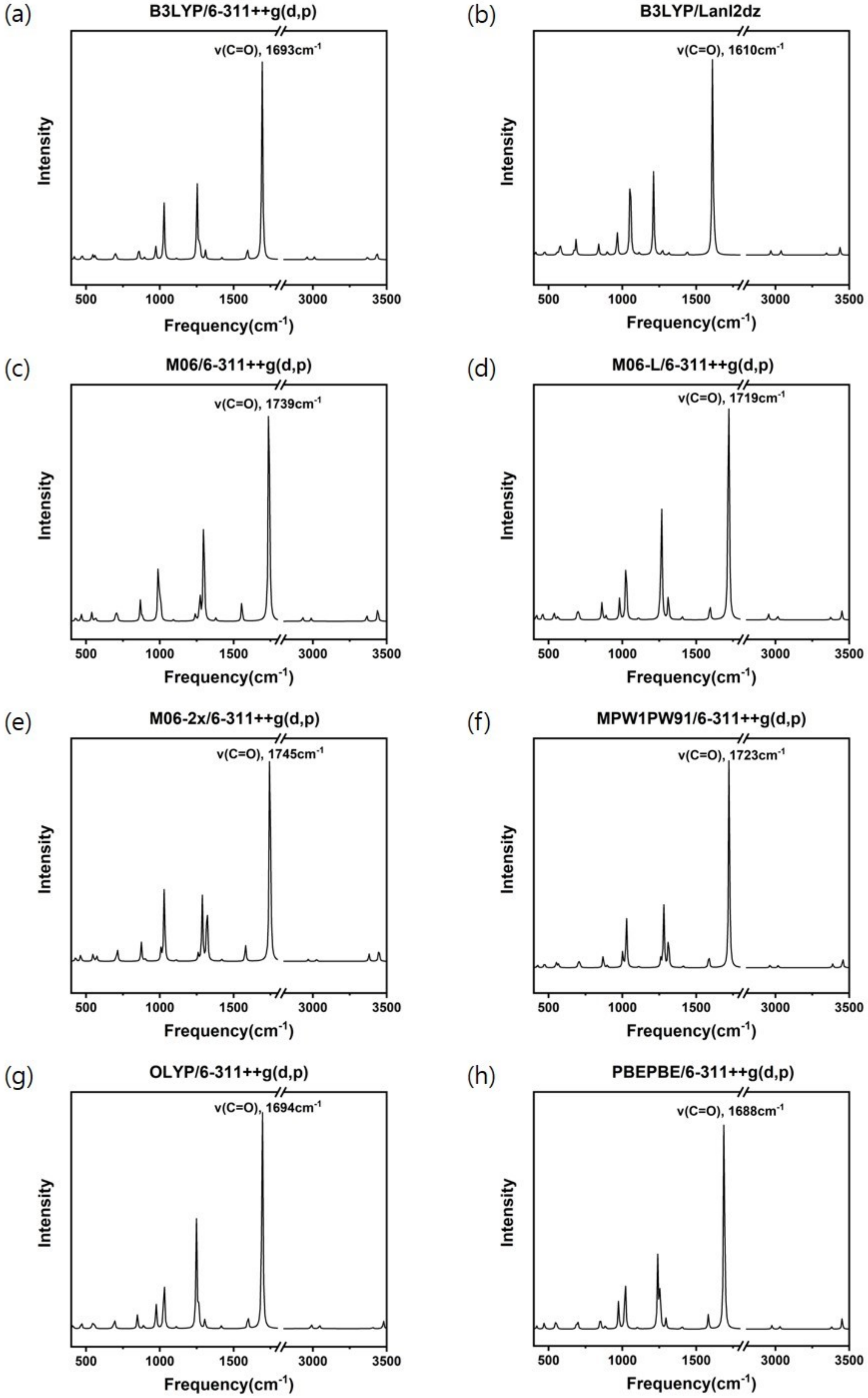

SI Fig. 2. IR spectra of single Zn-Gly_2

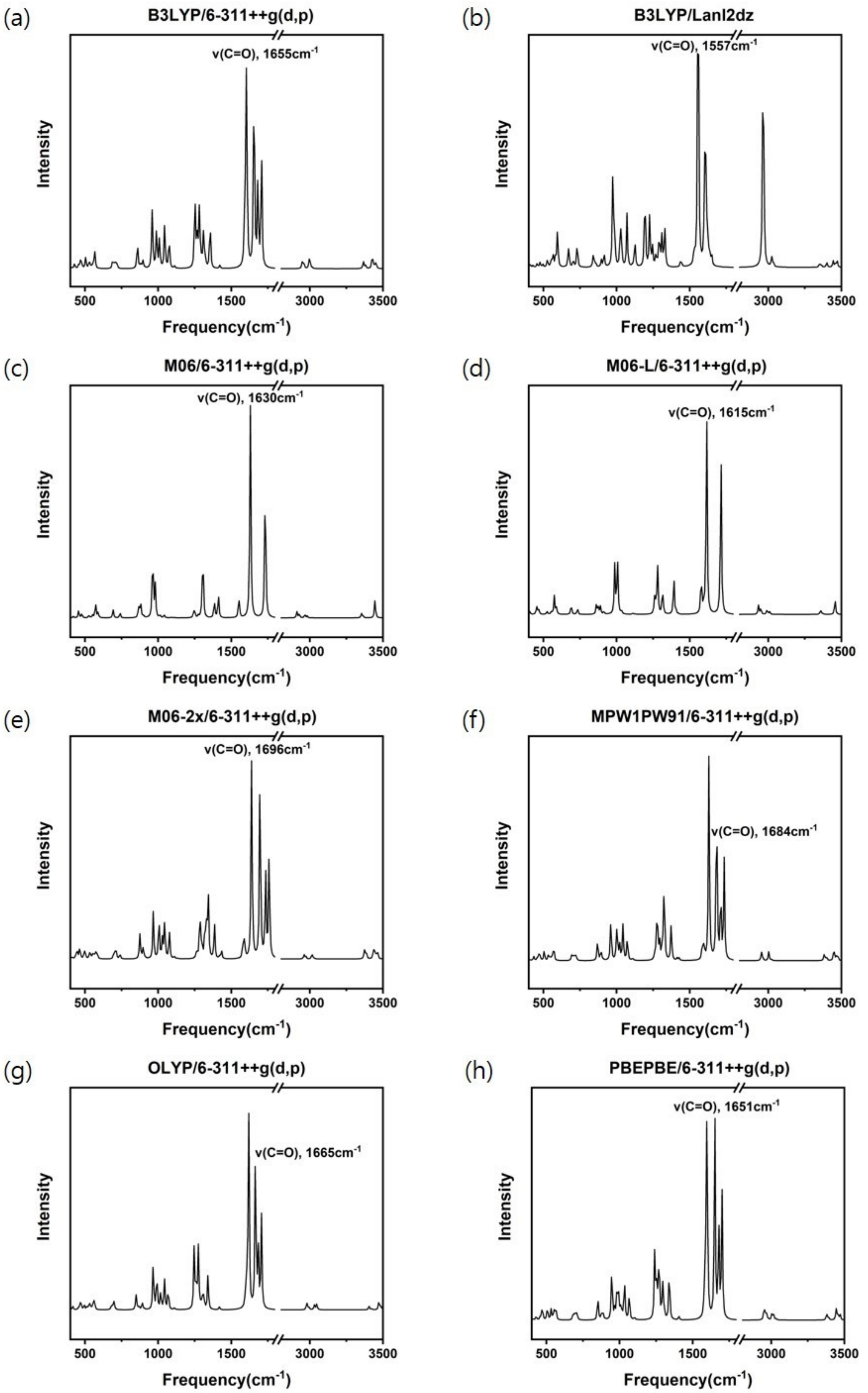

SI Fig. 3. IR spectra of single Zn-Met_2

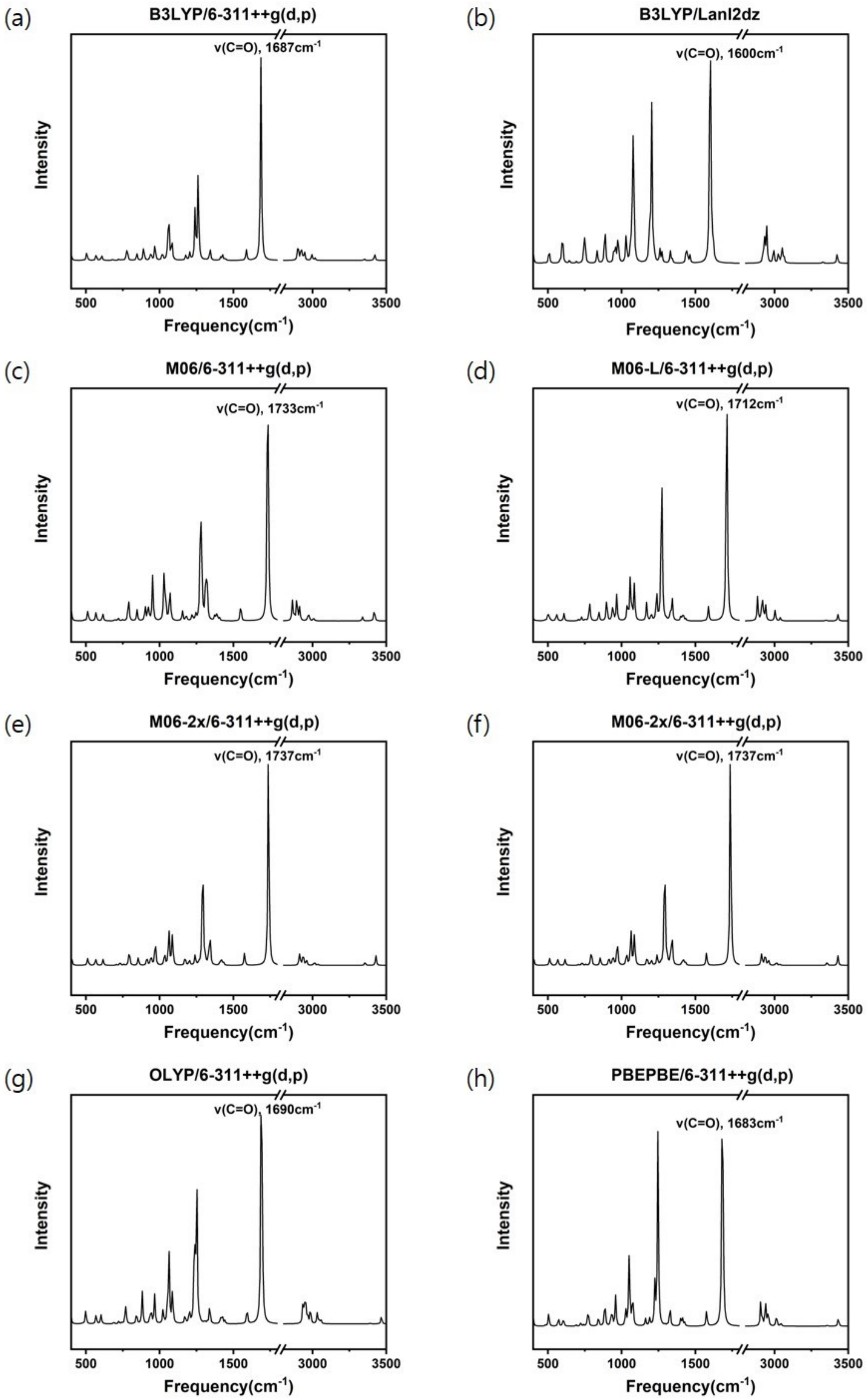

SI Fig. 4. IR spectrum of double Zn-Gly_2

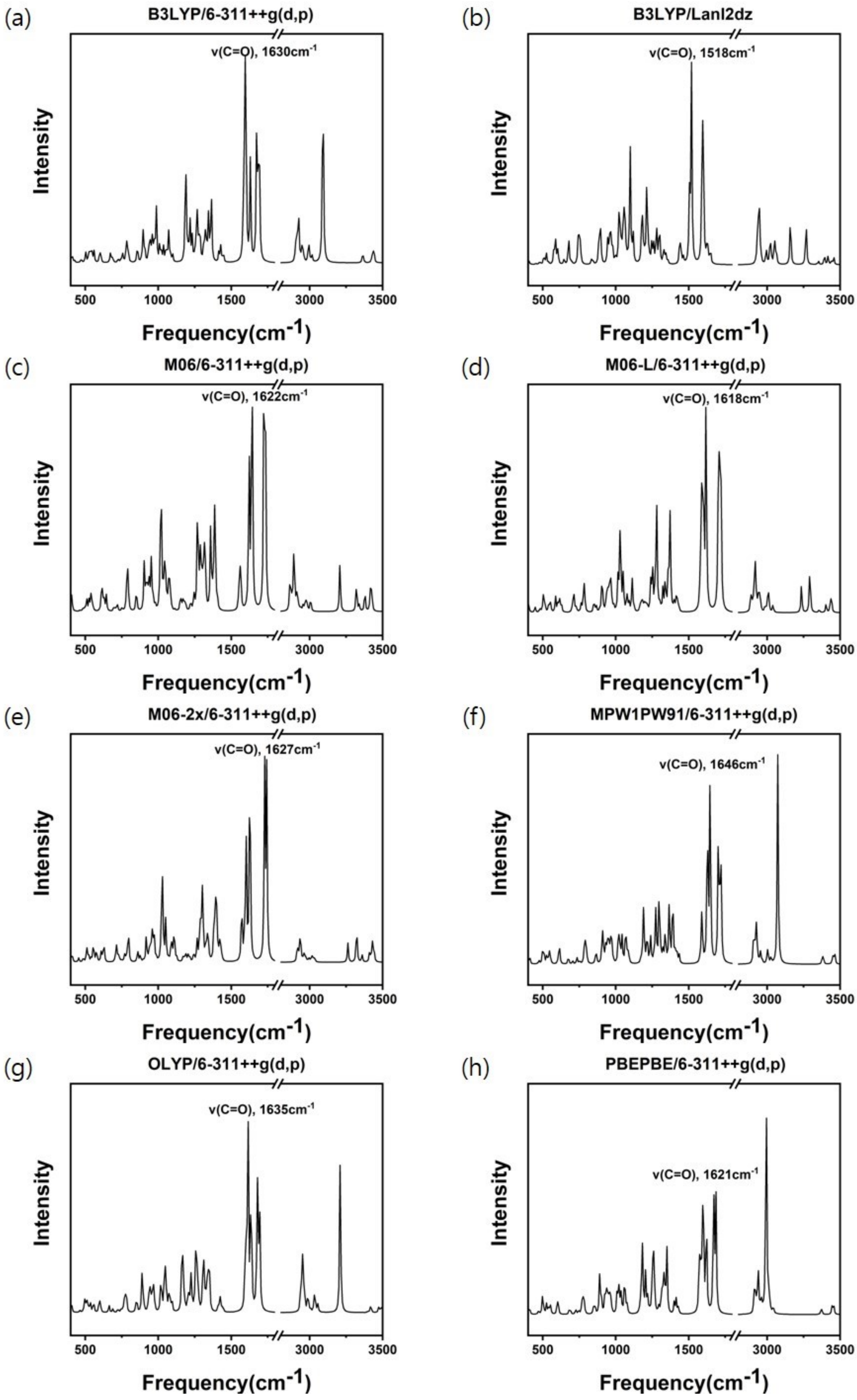

SI Fig. 5. IR spectra of double Zn-Met_2
